# Dual effectiveness of a novel all-in-one endodontic irrigating solution in antibiofilm activity and smear layer removal

**DOI:** 10.3389/fbioe.2023.1254927

**Published:** 2023-08-01

**Authors:** Xuyan Sheng, Jian Yu, He Liu, Zhejun Wang, Shuli Deng, Ya Shen

**Affiliations:** ^1^ Stomatology Hospital, School of Stomatology, Zhejiang University School of Medicine, Zhejiang Provincial Clinical Research Center for Oral Diseases, Key Laboratory of Oral Biomedical Research of Zhejiang Province, Cancer Center of Zhejiang University, Engineering Research Center of Oral Biomaterials and Devices of Zhejiang Province, Hangzhou, China; ^2^ Division of Endodontics, Department of Oral Biological and Medical Sciences, Faculty of Dentistry, University of British Columbia, Vancouver, BC, Canada; ^3^ State Key Laboratory of Oral and Maxillofacial Reconstruction and Regeneration, Key Laboratory of Oral Biomedicine Ministry of Education, Hubei Key Laboratory of Stomatology, School and Hospital of Stomatology, Wuhan University, Wuhan, China

**Keywords:** antimicrobial, biofilm, dentin, irrigant, smear layer

## Abstract

The continuous destruction of dental hard tissues increases the risk of bacterial invasion, which leads to pulp infections. Irrigation is critical for successful root canal treatment in terms of infection control. However, no single irrigant covers all of the functions demanded, including antibiofilm and tissue-dissolving activities. The aim of this study was to investigate the antimicrobial properties of Triton, an all-in-one irrigant, on *Enterococcus faecalis* and multispecies oral biofilms in dentin canals, as well as its ability to remove the smear layer. Dentin blocks (192 specimens) were prepared from single-root human teeth and then assigned to 48 groups (24 groups for each biofilm type). Serial centrifugation was used for bacterial introduction into dentinal tubules. After 3 weeks, half of the specimens were created a uniform smear layer. The following treatments were applied: short time (separate): Triton, 6% NaOCl, 2% NaOCl, and water (all for 3 min); short time (combined): Triton (3 + 1 min), 6% NaOCl +17% EDTA (3 + 1 or 2 + 1 min), and 2% NaOCl +17% EDTA (3 + 1 min); and long time: Triton (3 + 3 min), 6% NaOCl (5 min), 6% NaOCl +17% EDTA (5 + 1 min), and water (3 + 3 min). Confocal laser scanning microscopy and scanning electron microscopy were employed to examine the antimicrobial activity and smear layer removal, respectively. The results revealed that despite the absence or presence of the smear layer, Triton (3 + 3 min) showed the highest killing for both tested biofilms (61.53%–72.22%) among all groups (*p* < 0.05). Furthermore, the smear layer was removed by Triton after 3 + 3 min, exposing open dentin canals. These findings demonstrated that Triton can provide dual benefits of antibiofilm and smear layer removal capabilities simultaneously, indicating a simplified and effective strategy for application in root canal treatment.

## 1 Introduction

Human teeth are effective in combating external adverse challenges to protect the internal pulp tissues because of their delicate microstructure and ordered crystal arrangement (An et al., 2012). In nature, dental hard tissues are rarely self-repairing; the destruction of enamel and dentin allows bacteria to invade the pulp and cause infection and pain (Lawn et al., 2010; Yu et al., 2023). The primary purpose of endodontic treatment focuses on eradicating microorganisms within the infected root canals and preventing reinfection, thereby saving the natural tooth (Zehnder and Belibasakis, 2015). The necrotic, inflamed tissue/dentin debris and microbes inside the root canal can be removed under constant irrigation following mechanical instrumentation (Neelakantan et al., 2017). Irrigating solutions play a vital role in influencing canal wall areas that are not touched by the instruments (Boutsioukis and Arias-Moliz, 2022) and are beneficial in preventing bacterial extrusion into the periapical areas; their distinguishing features include organic or inorganic tissue-dissolving ability or antimicrobial/antibiofilm activities (Haapasalo et al., 2014). Despite advancements in disinfectants, instruments, and techniques have made endodontic treatment more predictable nowadays, there is no single irrigant that adequately covers all of the functions demanded. As a result, the combined, sequential use of irrigants is crucial to the clinical success of root canal treatment.

Sodium hypochlorite (NaOCl) is the most commonly used irrigant in endodontic treatment, due to its ability to dissolve organic tissues (including collagen, pulpal remnants, and organic components of the smear layer) and its broad-spectrum antimicrobial activity against *Enterococcus faecalis* (*E. faecalis*) and multispecies biofilms (Gu et al., 2017). Whereas, removal of dentin debris and inorganic components of the smear layer is also required for the complete cleaning of the root canal system (Bilvinaite et al., 2022). Ethylenediaminetetraacetic acid (EDTA) or citric acid (CA) is generally applied following NaOCl to effectively dissolve the inorganic substances of the smear layer and dentin (Turk et al., 2015). However, neither has any or little organic tissue-dissolving ability (Prado et al., 2015). Because of its favorable antibacterial properties, chlorhexidine digluconate (CHX) has long been employed in dental disinfection and plaque controlling (Kamolnarumeth et al., 2021). CHX, on the other hand, cannot replace NaOCl since it is incapable of dissolving organic debris and killing oral biofilms (Wang et al., 2020). Given that the successive use of these irrigants prolongs root canal cleaning, using combination products of irrigants with multiple functions would be desirable to simplify the clinical procedures.

The past few decades have witnessed the introduction of several combination products for root canal irrigation. Previous studies have shown that 2-in-1 solutions incorporating EDTA or CA and CHX or doxycycline can provide benefits (Andrabi et al., 2013; Balto et al., 2015; Giardino et al., 2018), but incorporating NaOCl in these solutions is not practical as EDTA neutralizes NaOCl (Rossi-Fedele et al., 2012). Additionally, concerns cover cytotoxicity, tooth staining, and drug resistance exist with the use of CHX or doxycycline (Shen et al., 2016; Liu et al., 2018; Wang et al., 2020). To address these concerns, a new all-in-one endodontic irrigant, Triton, has recently been developed, which avoids the use of EDTA, CHX, and antibiotics. Triton comprises two parts with distinct components, with Part A containing chelators (CA), surfactants, pH modifiers, and stabilizers, while Part B containing 8% NaOCl and a pH modifier. The automix technique precisely mixes the two parts to deliver the final solution (4% NaOCl), allowing for simultaneous organic and inorganic tissue dissolution. Despite its potential benefits, no related information on the antibiofilm activity and smear layer removal of Triton is currently available.

The objective of this study was to evaluate the antibiofilm and smear layer removal effectiveness of Triton as an endodontic irrigant. The null hypotheses were that: i) there is no significant difference in the antimicrobial efficacy between Triton and NaOCl or NaOCl + EDTA against *E. faecalis* and multispecies oral biofilms in dentin canals; and ii) Triton does not remove the smear layer.

## 2 Materials and methods

### 2.1 Dentin block preparation

**FIGURE 1:**
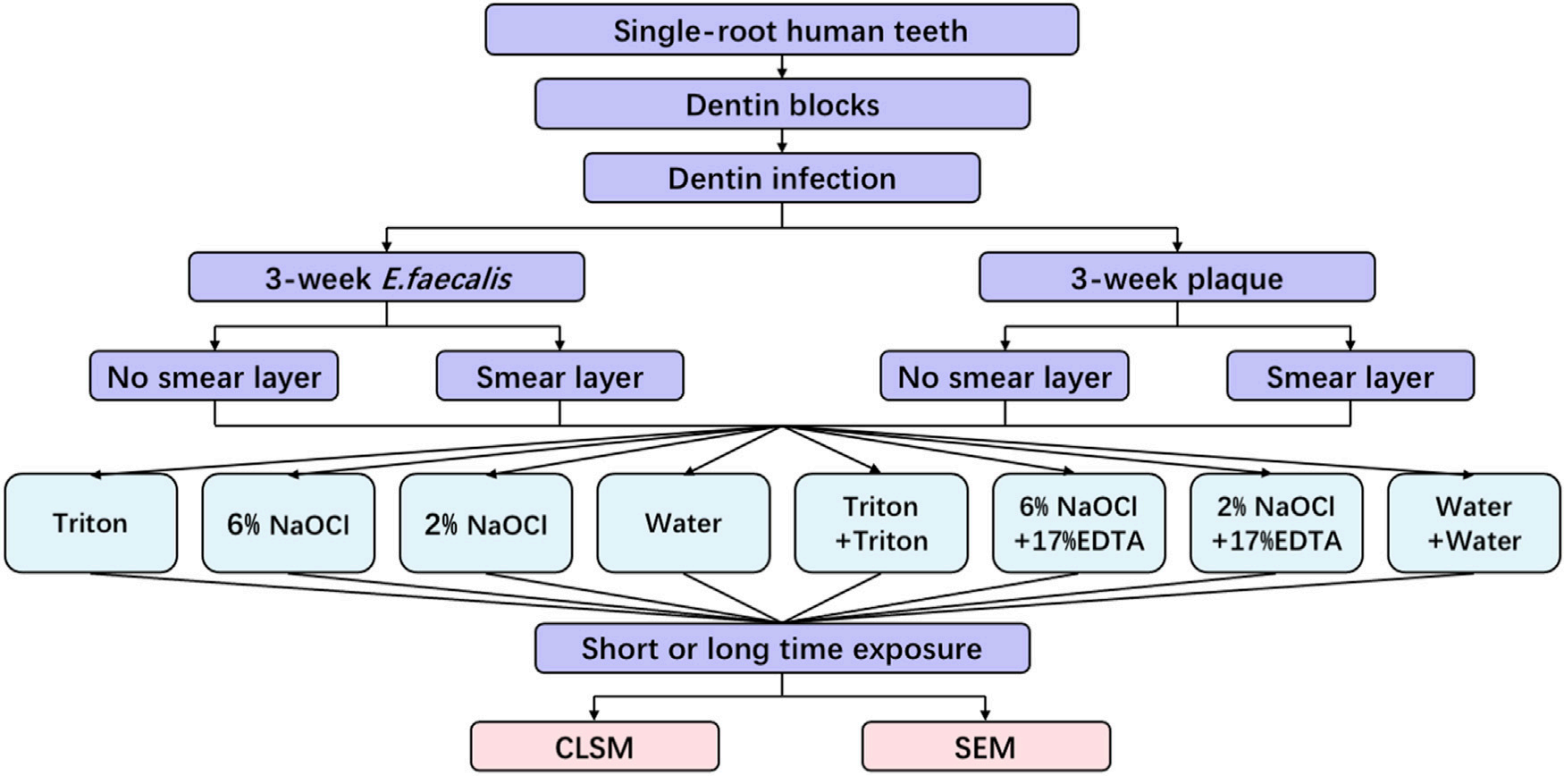
Schematic illustration of the experimental design in this study.

In accordance with the protocol approved by the Clinical Research Ethics Committee of the University of British Columbia (certificate H12-02430), 48 single-root, non-caries human teeth subjected to orthodontic extraction were collected. All procedures were performed following the Declaration of Helsinki. As the same procedure described in our previous studies (Wang et al., 2018; Huang et al., 2019), 96 dentin blocks (with a dimension of 4 × 4 × 2 mm) were prepared to yield 192 specimens. The schematic diagram of this study is presented in Figure 1.

### 2.2 Disinfecting solution preparation

Triton (Brasseler, Savannah, United States) was prepared by mixing components from Parts A and B at a 1: 1 volume ratio. The composition of ingredients of Triton is summarized in Supplementary Table S1. Seventeen percent EDTA (pH 7.0) (Vista, Racine, United States) and 6% NaOCl (Clorox, Oakland, United States) were achieved from the manufacturers. The iodometric titration was used to verify the available chlorine concentration. Sterilized deionized water was used to dilute 6% NaOCl for 2% NaOCl preparation.

### 2.3 Dentin block infection

The bacterial strain, *E. faecalis* VP3-181, was initially isolated from an infected root canal as previously reported (Peciuliene et al., 2000). Brain-heart infusion (BHI) agar (BD Corp., Sparks, United States) plates were used to anaerobically incubate *E. faecalis* overnight at 37°C. Supra- and subgingival plaque from one healthy adult volunteer was collected after informed consent was obtained. BHI broth was utilized to suspend *E. faecalis* and plaque mixed bacteria, respectively. In line with a previously published protocol (Ma et al., 2011), suspensions of *E. faecalis* and plaque were centrifuged into dentinal tubules after being adjusted to the same optical density (0.05 at 405 nm). A 3-week anaerobic incubation was implemented at 37°C for dentin blocks with bacterial suspensions in BHI broth. During the incubation period, fresh BHI broth was used to change the old broth once a week.

### 2.4 Dentin disinfection

After 3 weeks, the infected specimens were removed from the tubes. The nail varnish was employed to mimic the cement layer on the root surface by sealing the outer side of the specimens after sterile water rinse and air drying. According to different solutions and treatment times as shown in Table 1, the prepared specimens were randomly assigned to 48 groups (24 groups for *E. faecalis* and the other 24 groups for plaque; 4 specimens in each group). For groups with the smear layer, a medium-grit cylinder flat-end bur (Patterson Dental, Halifax, Canada) was used to create a smear layer on the root canal side of each block for 4 s each at 1,500 rpm prior to treatments. After that, 50 μL of each solution was applied on the root canal side of the specimens for designated short and long periods of time. To expose fresh surfaces of longitudinally fractured dentinal tubules (Wang et al., 2017), each specimen was vertically split across the center of the root canal into two-halves after rinsing for 1 min with sterile water. Each specimen in the groups treated with two solutions was rinsed with PBS for 30 s and dried before being treated with the second solution.

**TABLE 1:** Times of exposure to the experimental solutions used in this study.

Solutions	Short time (min)		Long time (min)	
	No smear layer	Smear layer	No smear layer	Smear layer
**Triton**	3	3	─	─
**6% NaOCl**	3	3	5	5
**2% NaOCl**	3	3	─	─
**Water (control)**	3	3	─	─
**Triton + Triton**	3 + 1	3 + 1	3 + 3	3 + 3
**6% NaOCl + 17% EDTA**	3 + 1/2 + 1	3 + 1/2 + 1	5 + 1	5 + 1
**2% NaOCl + 17% EDTA**	3 + 1	3 + 1	─	─
**Water + Water (control)**	─	─	3 + 3	3 + 3

### 2.5 Examination of confocal laser scanning microscopy (CLSM)

Following gentle rinsing with PBS for 1 min, each specimen was stained with a live/dead bacterial viability kit (L7012, Molecular Probes, Eugene, United States) protected from light according to the manufacturer’s protocols. SYTO-9 and propidium iodide included in this kit enable live and dead bacteria to show green and red fluorescence, respectively (Huang et al., 2019; Yu et al., 2021). CLSM (FV10i-LIV, Olympus, Tokyo, Japan) was employed to scan biofilm images (512 × 512 pixels) from four randomly selected areas for each specimen with a z-stack of 20 slices at 0.5-μm step, and a minimum of 16 scans were obtained for each group. An Imaris 7.4.2 software (Bitplane, Zurich, Switzerland) was used to reconstruct three-dimensional volume stacks and quantitatively determine the proportions of live and dead bacteria (Guo et al., 2021; Zhu et al., 2023).

### 2.6 Smear layer removal

Additional dentin disk specimens were prepared in accordance with previously proposed approach (Wang et al., 2017). These specimens were exposed to one of five freshly prepared solutions (2 mL) as follows: i) 6% NaOCl +17% EDTA (3 + 1 min), ii) 6% NaOCl +17% EDTA (5 + 1 min), iii) 6% NaOCl + water (5 + 1 min), iv) Triton (3 min), and v) Triton + Triton (3 + 3 min). This process was conducted with gentle vibration (60 rpm) at room temperature. Sterilized deionized water was used to rinse the specimens for 1 min between exposure to two solutions, and a final rinse for 1 min was applied for all specimens. Scanning electron microscopy (SEM, SU3500, Hitachi, Toronto, Canada) was utilized to observe the smear layer removal at 3 kV.

### 2.7 Colony forming unit (CFU) test

Sterile hydroxyapatite (HA) disks (Clarkson Chromatography Products, Williamsport, United States) with 1.52 mm thickness and 9.65 mm diameter were used as the growth substrates of plaque biofilms. The formation of biofilms on HA disks was performed based on a well-established model, as previously reported (Wang et al., 2020). The 3-week-old biofilms formed on the surface of disks were scraped off into BHI broth medium, and the suspension was adjusted to an optical density of 0.25 at 405 nm. One hundred μL of each plaque suspension was then added to 400 μL of sterile water, 6% NaOCl, and Triton for 30 s. After that, 100 μL of each suspension was added to 900 μL of BHI broth medium to perform ten-fold serial dilution. A droplet of 20 μL of diluent from each of three solutions was overspread onto blood agar plates (BHI agar with 5% heparinized sheep’s blood; BD Difco, Detroit, United States) and anaerobically cultivated at 37°C for 48 h, followed by calculating the total number of CFU. The percentage of bacteria killed was defined as previously described (Wang et al., 2020). Three repeated tests were accomplished for the CFU test.

### 2.8 Statistical analysis

Statistical analysis was carried out using SPSS 22.0 (IBM, Armonk, United States). The Shapiro-Wilk test and Levene’s test were utilized to confirm the normality of the distribution and the homogeneity of variance, respectively. Univariate analysis of variance (ANOVA) with *post hoc* multiple comparisons was employed to analyze the CLSM and CFU data. The significance level was set at 0.05.

## 3 Results

### 3.1 Antimicrobial effects by CLSM analysis

**FIGURE 2:**
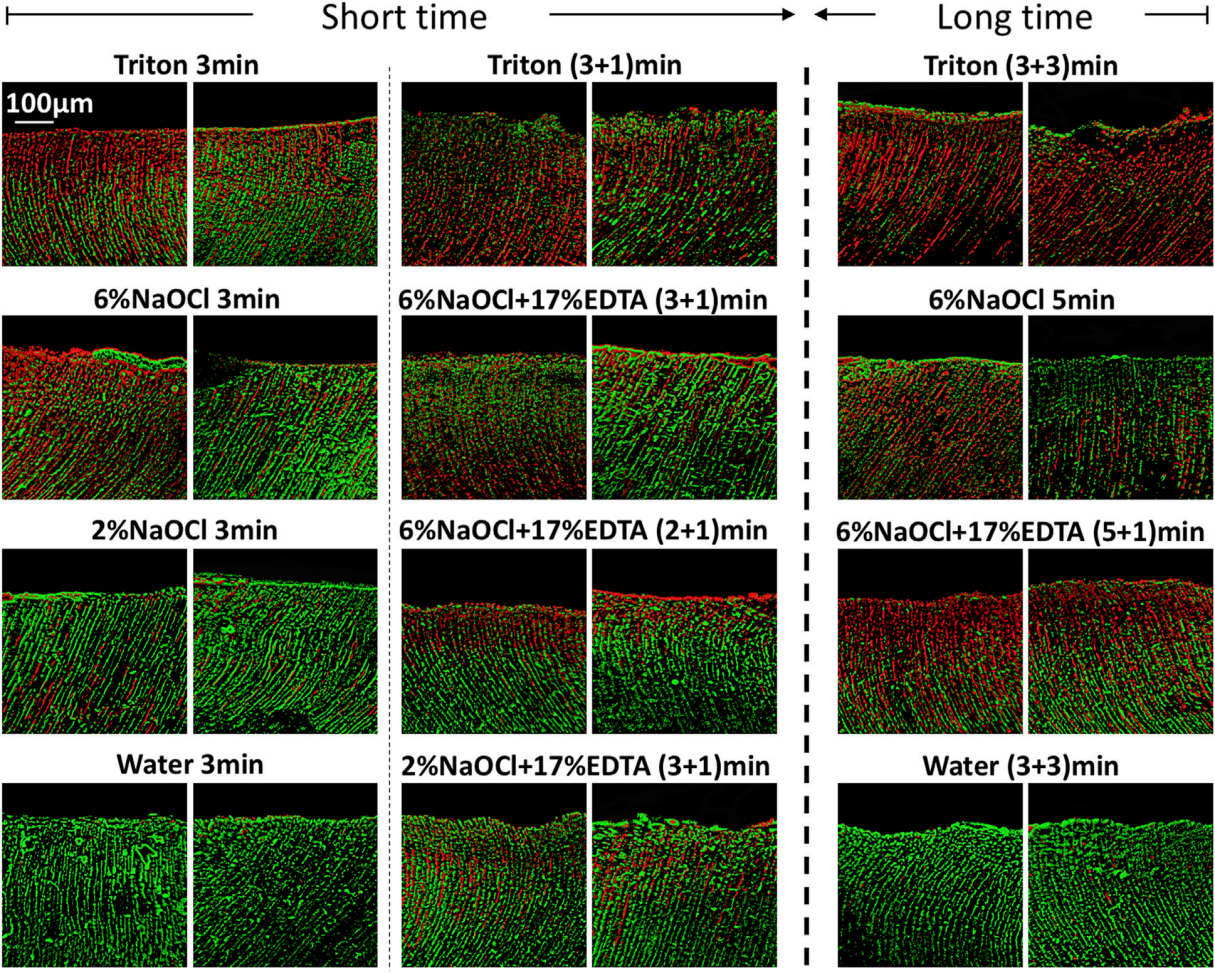
Three-dimensional reconstructed CLSM images of 3-week-old *Enterococcus faecalis* biofilms in dentin canals after exposure to different irrigants (live bacteria, green; dead bacteria, red). For each medication, pictures on the left and right respectively show the absence and presence of the smear layer.

**FIGURE 3:**
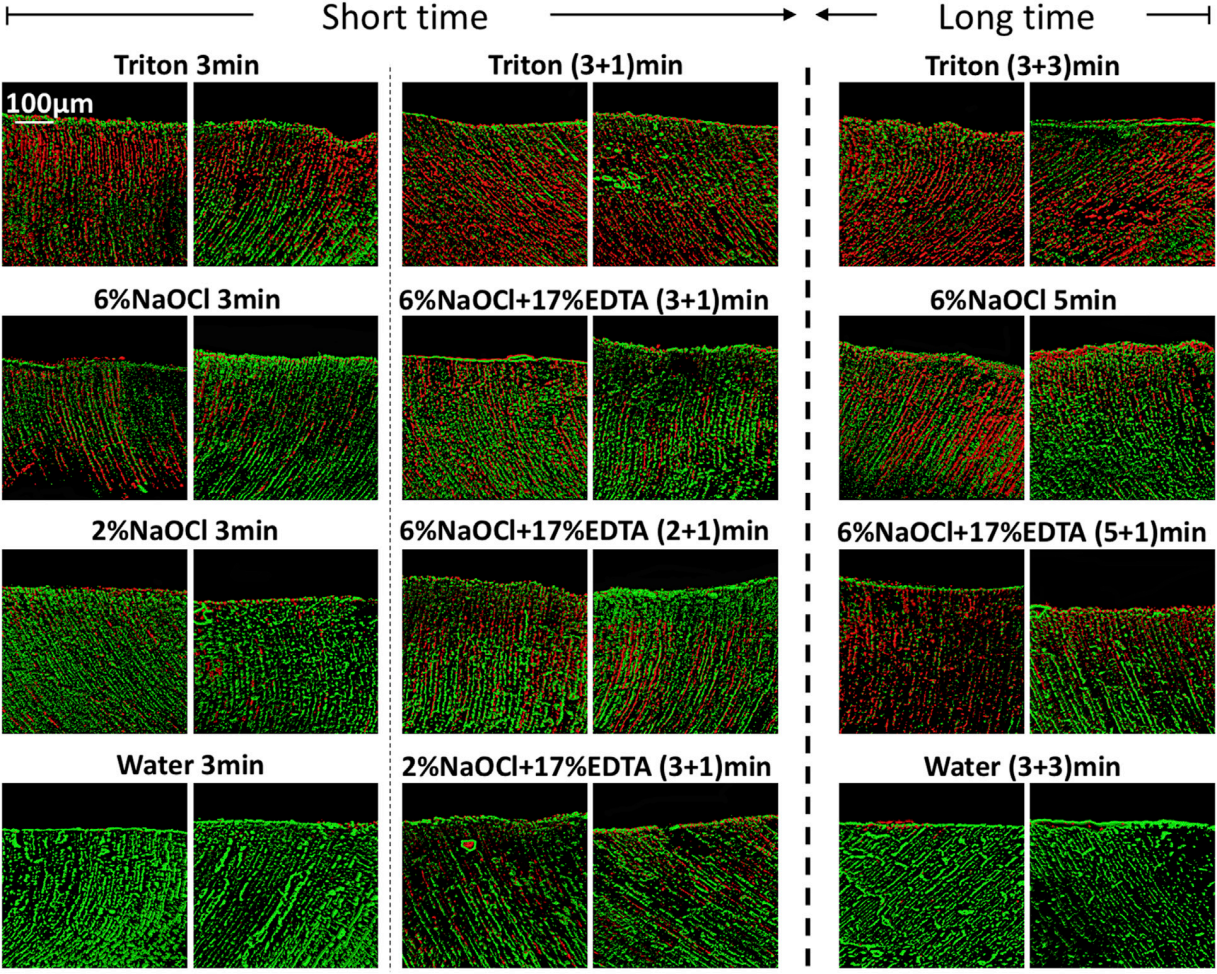
Three-dimensional reconstructed CLSM images of 3-week-old plaque multispecies biofilms in dentin canals after exposure to different irrigants (live bacteria, green; dead bacteria, red). For each medication, pictures on the left and right respectively show the absence and presence of the smear layer.

**FIGURE 4:**
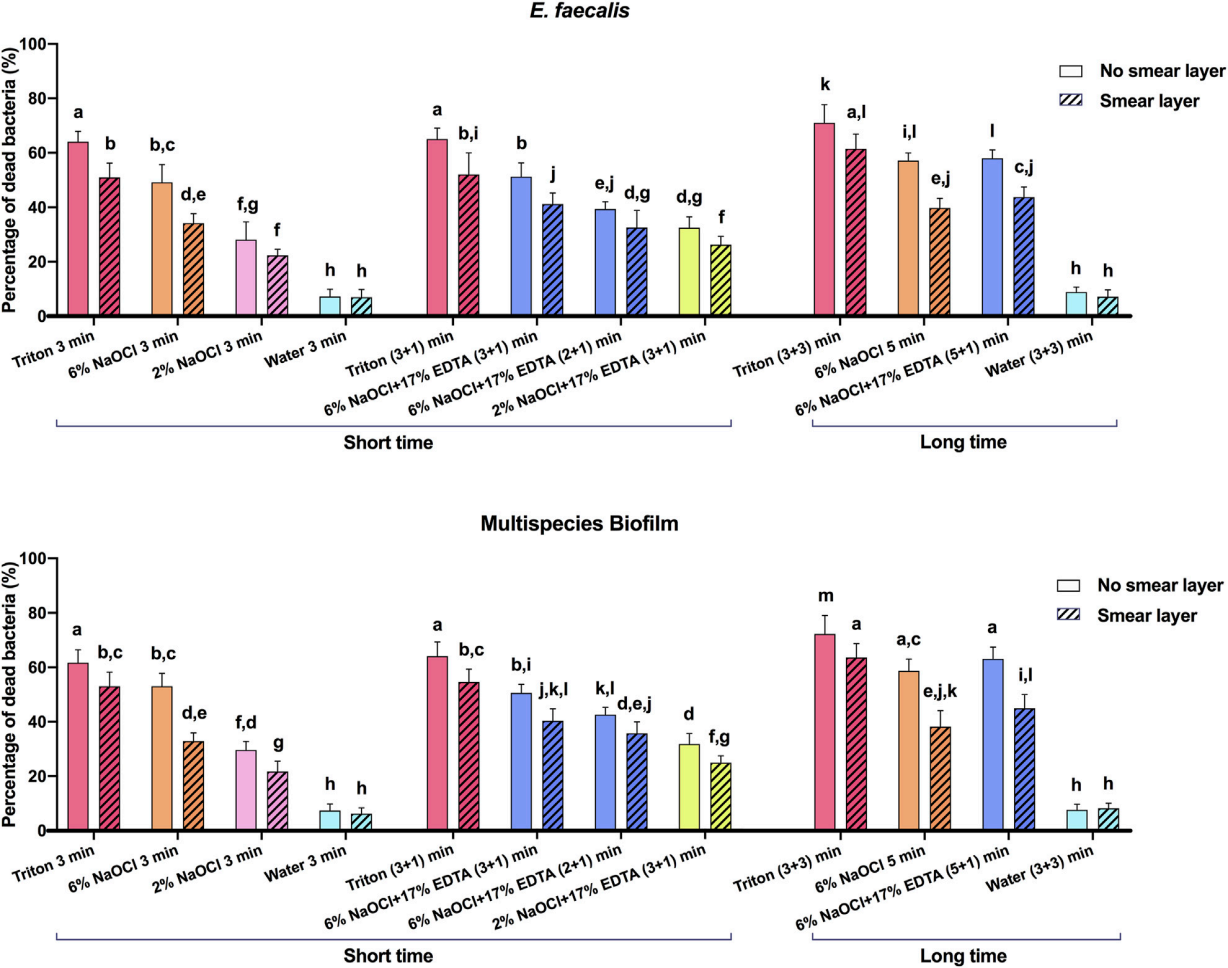
The proportions of dead bacterial cell volumes in 3-week-old biofilms of dentin canals after short and long exposure to different irrigants. Data are represented as means ± standard deviations. Different letters indicate statistical differences (*p* < 0.05).

CLSM images of 3-week-old *E. faecalis* and plaque multispecies biofilms in dentin canals after exposure to different irrigants are shown in Figure 2 and Figure 3, respectively, and the corresponding proportions of dead bacterial cells in the biofilms are summarized in Figure 2. In the absence of the smear layer (Figures 2–4), large proportions of two types of bacteria (64.09% ± 3.81% for *E. faecalis* and 61.73% ± 4.72% for multispecies biofilms) were killed by Triton alone in 3 min, while 6% NaOCl induced approximately half of the bacterial death. The use of Triton by 3 + 3 min killed 71.05% ± 6.68% in *E. faecalis* and 72.22% ± 6.81% in multispecies biofilms, achieving the highest biofilm killing among all groups (*p* < 0.05), irrespective of bacterial type or short or long exposure.

In the presence of the smear layer (Figures 2–4), Triton alone was as effective as 6% NaOCl (without the smear layer) in killing *E. faecalis* and multispecies biofilms in 3 min, while outperforming 6% NaOCl (with the smear layer) (*p* < 0.05). Six percent or 2% NaOCl +17% EDTA was more effective in bacterial killing than NaOCl alone, and the weakest effects were found when 2% NaOCl was used alone. Long exposure to Triton by 3 + 3 min resulted in significantly higher bacterial death for both *E. faecalis* (61.53% ± 5.36%) and multispecies (63.65% ± 5.05%) biofilms, when compared to all other groups (*p* < 0.05).

### 3.2 Smear layer removal

**FIGURE 5:**
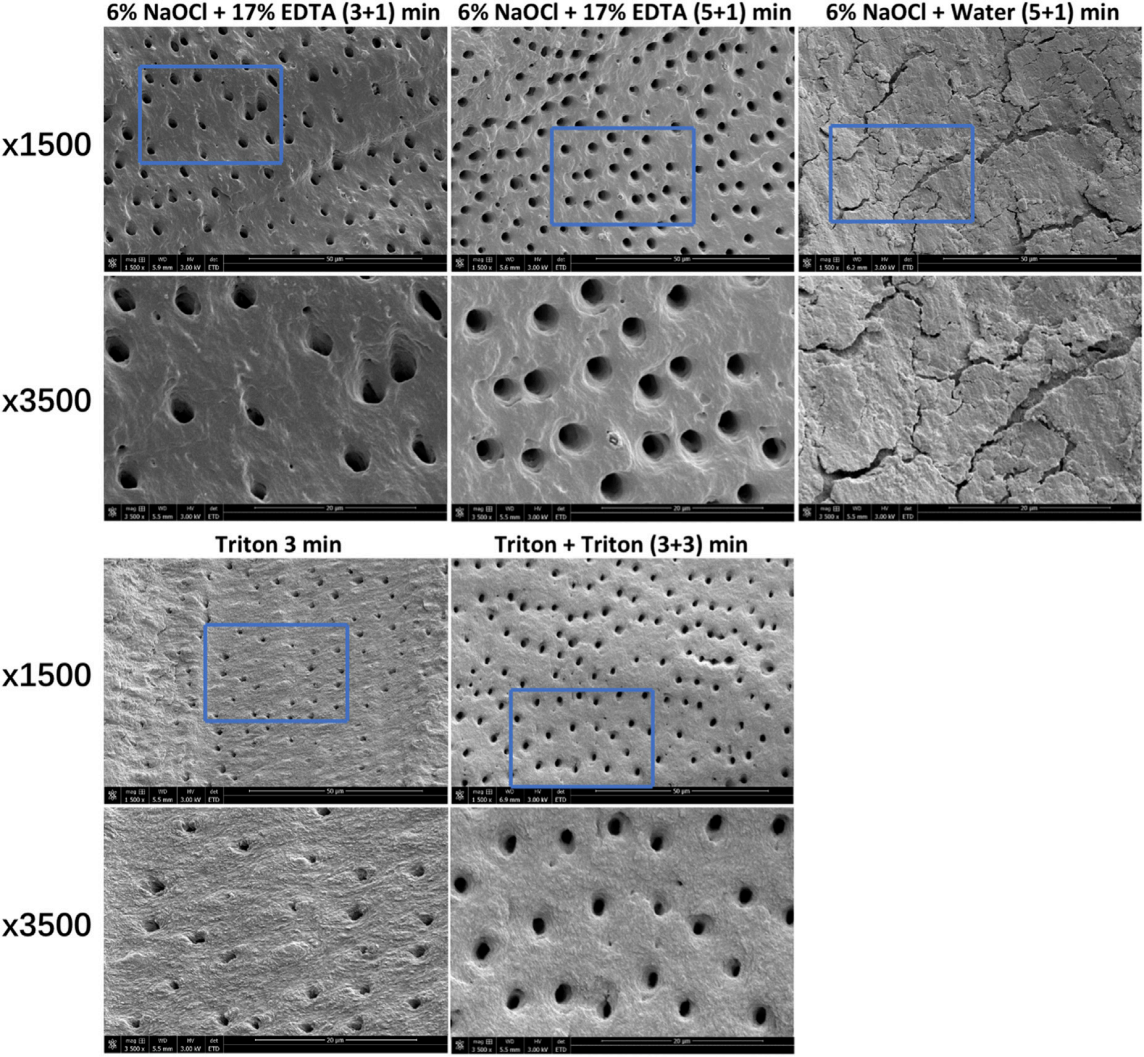
Characteristic SEM images of smear layer removal by different treatments. For each group, images (×3,500) correspond to high-magnification images of the blue box region (1,500 ×).

Figure 5 shows the typical SEM images of smear layer removal by different treatments. Groups containing Triton or EDTA removed the smear layer to varying degrees, leaving open dentin canals exposed. In contrast, the 6% NaOCl + water (without EDTA) group did not cause the removal of the smear layer, and no open dentinal tubules were visible.

### 3.3 Bacterial killing by CFU test

Supplementary Figure S1 displays CFU results for 3-week-old dispersed plaque multispecies biofilms after exposure to different medicaments for 30 s. The use of 6% NaOCl killed 99.46% ± 0.15% of the bacteria. As for Triton, all of the bacteria were killed.

## 4 Discussion

The effectiveness of Triton on killing mono- and multispecies oral biofilms and removing the smear layer in dentin canals was evaluated in the present study. The results demonstrated that Triton showed significantly higher bacterial killing for both *E. faecalis* and multispecies biofilms in dentin canals when compared to NaOCl or NaOCl + EDTA and can remove the smear layer with open dentin canals exposed. As a result, Triton is effective in providing the antibiofilm and smear layer removal activities, and the two null hypotheses have to be rejected.

The prerequisite for successful endodontic treatment derives from effective bacterial biofilm elimination in the root canal systems. Both *E. faecalis* and multispecies biofilms were tested since the former is generally detected in chronic endodontic infection and the latter could better mimic actual oral conditions (Shen et al., 2011; Rosa et al., 2017). Treatment with Triton for 3 min resulted in significant bacterial killing in both biofilms (Figures 2–4). Interestingly, when Triton was used for 3 min, followed by another 3 min of treatment, it showed significantly higher biofilm bacterial death compared to 6% NaOCl (5 min) and 6% NaOCl + 17% EDTA (5 + 1 min), regardless of the presence of the smear layer. It is worth noting that clinical cases may involve more than one canal in a tooth, potentially necessitating longer irrigant exposure. Therefore, both short-time and long-time irrigation were employed in this study to account for such scenarios. The findings suggest that Triton exhibits superior antimicrobial effects on oral mono- and multispecies biofilms within dentin canals, outperforming NaOCl or NaOCl + EDTA. Additionally, the long-time treatment with Triton resulted in even more pronounced antimicrobial effects compared to the short-time treatment. These results underscore the potential of Triton as an effective antimicrobial agent for endodontic procedures. Unlike conventional irrigants or 2-in-1 products, Triton avoids the use of EDTA and/or CHX to work differently because NaOCl neutralizes upon contact with EDTA rapidly. Two separate components are designed in Triton, and the automix mechanism will precisely mix Part A (contains CA and surfactants) and Part B (contains 8% NaOCl) to produce the final solution (4% NaOCl). Interestingly, Triton with 4% NaOCl had higher antibiofilm activity than 6% NaOCl alone or in combination with 17% EDTA. The reasons may ascribe to the following perspectives. CA may promote the antibiofilm effectiveness of hypochlorite in deeper layers of dentin with the removal of the smear layer (Campello et al., 2022). Previous studies have highlighted that the antimicrobial and tissue-dissolving effects of hypochlorite also can be enhanced by the addition of surfactants (Stojicic et al., 2010; Iglesias et al., 2019). It is worth mentioning that differences in solution concentrations and tissue types may lead to partially contradictory outcomes (Poggio et al., 2010; Clarkson et al., 2012). Furthermore, pH modifiers (specifically sodium hydroxide) contained in Triton could maintain NaOCl solutions at an alkaline pH. As a result, the alkaline environment inside dentin canals is beneficial to combat acidic by-products generated by microbial metabolism within biofilms to enhance the cleansing effects (Ran et al., 2015).

In addition to eradicating biofilms in root canal systems, the dissolution of necrotic, infected organic and inorganic substances with irrigating solutions is required for complete cleaning. The smear layer is typically created during instrumentation and may act as an impediment to microbial killing of antimicrobial agents in the canals (Wang et al., 2013). In this regard, it is crucial that the smear layer should be removed. Since hypochlorite can only remove the organic components of the smear layer, the activity of irrigants against inorganic components and dentin debris is preferred. Hence, the ability of Triton on the smear layer removal was determined in this study. SEM observation confirmed that Triton treatment for 3 and 3 + 3 min removed the smear layer with open dentin canals exposed (Figure 5), which is as effective as NaOCl + EDTA, implying a favorable tissue-dissolving activity. This finding could be related to the function of CA incorporated in Triton. Despite the scarce antibacterial activity, CA as a chelator is capable of effectively dissolving inorganic materials, including dentin remnants and apatite crystals (Gandolfi et al., 2018). Recent literature has reported that synergistic action of mode interacting with other chemicals can be provided by CA (Olivieri et al., 2016; Wilkoński et al., 2021). Furthermore, the stable, high pH condition of NaOCl solutions created by pH modifiers plays a vital role in exerting stronger proteolytic effects. Significantly more necrotic, inflamed tissue, dentin debris, and inorganic components of the smear layer are likely to be dissolved according to previous evidence (Jungbluth et al., 2011; Trautmann et al., 2021). In other words, the all-in-one design in Triton demonstrated positive effects for the combination of NaOCl, CA, surfactants, and pH modifiers. It should be emphasized that the use of Triton did not weaken or compromise the efficacy of any of the components included. Instead, effective antimicrobial and tissue-dissolving activities can be achieved simultaneously.

As a critical field of research in endodontics, ideal dentin disinfection has been pursued for decades. The role of microorganisms in dentin canals may vary in different cases (Bergenholtz, 2016). In clinical practice, the effectiveness of various antimicrobial strategies can be considered one of the most useful indicators for dentin disinfection (Han et al., 2021; Yan et al., 2022; Hu et al., 2023). To simulate the clinical conditions more realistically in this study, short and long time exposure to different irrigating solutions was evaluated on the basis of previous investigations (Wang et al., 2018; Huang et al., 2019). Although it appears to be a relatively long irrigation exposure of 3 + 3 or 5 + 1 min, the significance is that searching for potential curved, calcified, or missing canals in multirooted teeth cases that require a prolonged time is available. Additionally, serial centrifugation for dentin blocks was implemented by introducing *E. faecalis* and multispecies bacteria into the dentinal tubules in our model. In this regard, it will be possible to make comparisons between specimens since all dentin blocks secure the bacteria inside the dentinal tubules via centrifugation (Ma et al., 2011; Al-Zuhair et al., 2023).

The application of Triton succeeds in the synergistic killing of oral bacterial biofilms and removal of the smear layer as an alternative irrigant. The action of mode of the all-in-one strategy also simplifies procedural steps for endodontic treatment. However, the limitations of this study should not be omitted. Given the complexity of dentin canal and apical structure, an *ex vivo* model (instead of an *in vitro* model) of dentin infection should also be employed to investigate the antimicrobial efficacy of the irrigants in future studies. Although promising findings have been demonstrated, it should be noted that the *in vitro* conditions tested in the present study may not exactly mimic actual oral conditions. In addition, to further determine the molecular biological mechanism of antibiofilm activity, additional analyses, such as polymerase chain reaction assay, need be considered apart from CLSM examination. Further research is also demanded to investigate the detailed mechanism and wide-ranging application of Triton.

## 5 Conclusion

The use of Triton effectively killed *E. faecalis* and multispecies oral biofilms in dentin canals and demonstrated a higher killing ability than 6% NaOCl and 6% NaOCl +17% EDTA. Meanwhile, Triton can be used to remove the smear layer while leaving the open dentinal tubules exposed. The all-in-one design for offering dual effectiveness of antibiofilm and smear layer removal capabilities indicates a promising strategy to optimize the mode of action of irrigation in root canal treatment. Thus, the development of Triton may serve as an alternative irrigant to provide significant benefits in leading a simplified and effective irrigation era in clinical practice.

## Data Availability

The original contributions presented in the study are included in the article/Supplementary Material, further inquiries can be directed to the corresponding authors.
