# Supplementary material for: Dual effectiveness of a novel all-in-one endodontic irrigating solution in antibiofilm activity and smear layer removal
Source: Front Bioeng Biotechnol. 2023 Aug 1;11:1254927. doi: 10.3389/fbioe.2023.1254927 (PMC10427723; doi:10.3389/fbioe.2023.1254927)
Supplement: Supplementary file 1 [file DataSheet1.PDF]

Supplementary Material

Dual effectiveness of a novel all-in-one endodontic irrigating solution in antibiofilm activity and smear layer removal

Xuyan Sheng[1,2,†], Jian Yu[2,3,†], He Liu[2], Zhejun Wang[2], Shuli Deng[1*], Ya Shen[2*]

[1]Stomatology Hospital, School of Stomatology, Zhejiang University School of Medicine, Zhejiang Provincial Clinical Research Center for Oral Diseases, Key Laboratory of Oral Biomedical Research of Zhejiang Province, Cancer Center of Zhejiang University, Engineering Research Center of Oral Biomaterials and Devices of Zhejiang Province, Hangzhou, China

[2]Division of Endodontics, Department of Oral Biological and Medical Sciences, Faculty of Dentistry, University of British Columbia, Vancouver, Canada

[3]State Key Laboratory of Oral & Maxillofacial Reconstruction and Regeneration, Key Laboratory of Oral Biomedicine Ministry of Education, Hubei Key Laboratory of Stomatology, School & Hospital of Stomatology, Wuhan University, Wuhan, China

*** Correspondence:**
Ya Shen; Shuli Deng
yashen@dentistry.ubc.ca; ytys090116@zju.edu.cn

[†]Xuyan Sheng and Jian Yu contributed equally to this work and designated as co-first authors.

Supplementary TABLE S1. Information on ingredients of the all-in-one irrigant (Triton) in this study.

Identification	Name	Percentage (%)
Mixture Part A	1,2,4-Butanetricarboxylic acid, 2-phosphono-	≤ 15
	Citric acid	≤ 2
	Sodium dodecylbenzenesulfonate	≤ 2
	Alcohols, C9-11, ethoxylated, liquids	≤ 2
	Polyethylene glycol 4-(tert-octylphenyl) ether	≤ 2
	Sodium lauryl sulfate	≤ 2
	2-Ethylhexyl sodium sulfate	≤ 2
	Sodium cumenesulphonate	≤ 2
	Sodium hydroxide	≤ 1
Mixture Part B	Sodium hypochlorite	≤ 10
	Sodium hydroxide	≤ 2

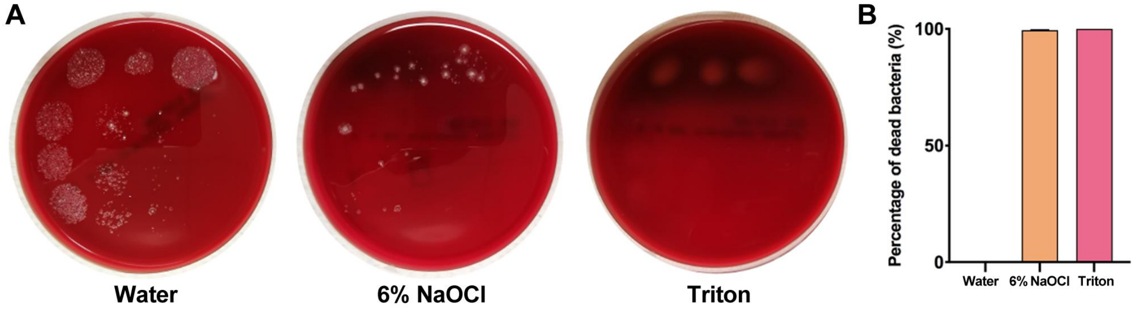

Supplementary Figure S1. Bacterial killing by CFU test for 3-week-old dispersed plaque multispecies biofilms after exposure to different solutions. (A) Pictures of colonies grown on BHI agar plates and (B) percentage of dead bacteria (%).
